# Developmental Changes in the Association Between Heart Rate Variability and Peer Success Across Early Elementary School

**DOI:** 10.1111/psyp.70156

**Published:** 2025-09-23

**Authors:** Danielle R. Rice, Lisa M. Gatzke‐Kopp, Zachary Fisher

**Affiliations:** ^1^ Department of Human Development and Family Studies The Pennsylvania State University University Park Pennsylvania USA

**Keywords:** biological sensitivity to context, emotion regulation, heart rate variability, teacher–child relationships

## Abstract

Associations between higher baseline heart rate variability (HRV) and better emotion regulation (ER) outcomes are commonly observed among adolescents and adults, but are less consistent among children. It is possible that this association is developmentally emergent, but unclear whether baseline HRV reflects a different functional process in childhood. One possible function could be regulating individuals' openness or susceptibility to environmental influences. The present study tested the effects of a three‐way interaction among baseline HRV, teacher–child closeness, and school year (kindergarten, first grade, and second grade) on peer success in a sample of 339 children (*M*_age_ = 66.32 months, 70.2% Black, 64.3% male). We examined associations with peer success as indices of age‐appropriate development of ER. Two theoretical frameworks were tested developmentally: (1) an ER framework where high baseline HRV would relate directly to greater peer success, and (2) an environmental sensitivity (ES) framework where high baseline HRV would have positive effects on peer success in the context of a positive environment (i.e., high teacher–child closeness), but negative effects in the context of a negative environment (i.e., low teacher–child closeness). Results in kindergarten were partially consistent with the ES framework, whereas in first grade, results tentatively reflected the ER framework. Results suggest that the functional implications of baseline HRV may need to be considered in a developmental context.

## Impact Statement

Our findings extend what we know about the developmental functions of heart rate variability (HRV) in childhood, which contrast with those in adolescence and adulthood. These functional changes have implications for children's socioemotional adjustment, such that baseline HRV indicates environmental sensitivity (ES) in younger children but may better index emotion regulation (ER) as children get older.

## 1 Introduction

Heart rate variability (HRV), an index of parasympathetic regulation of cardiac activity, has been widely studied as a physiological correlate of psychopathology. Robust associations between low baseline HRV and an array of psychiatric diagnoses have led to the postulation that low baseline HRV reflects a weakened capacity to regulate emotional arousal and may be a transdiagnostic component of psychopathology (see Beauchaine 2015). Despite extensive support for this postulation in studies with adolescent and adult samples, studies involving children are far less consistent in detecting associations with baseline HRV (e.g., Calkins et al. 2007; Hastings et al. 2008). This discrepancy calls attention to the need to consider how psychophysiological markers relate to children's socioemotional functioning, with implications for the development of psychopathology (Trentacosta and Shaw 2009).

### 1.1 Baseline HRV and Children's Emotion Regulatory Capacity

Prominent theories of HRV center on the role of the parasympathetic nervous system in regulating arousal by exerting an inhibitory or decelerating influence on the heart, resulting in greater variability in heart rate across the respiratory cycle (sometimes referred to as respiratory sinus arrhythmia [RSA]). According to Polyvagal Theory, high baseline HRV is thought to reflect greater parasympathetic control, and thus greater capacity to regulate affective arousal through dynamic changes in parasympathetic input that can be titrated to situational demands (Porges 2007). Other frameworks like the Neurovisceral Integration Model similarly posit that high baseline HRV reflects activation of the central autonomic network (which includes brain structures such as the prefrontal cortex, amygdala, and hypothalamus) to help integrate information and execute stronger affective and, in turn, behavioral control (Thayer et al. 2009). Both frameworks effectively conceptualize baseline HRV as an index of emotion regulatory capacity (i.e., one's flexibility in responding to environmental demands and ability to apply emotional resources to navigate situations) and are consistent with findings of low baseline HRV across a range of psychiatric conditions characterized by emotion regulation (ER) deficiencies, including anxiety disorders, depression, borderline personality disorder, and antisocial behaviors (Cheng et al. 2022; Koch et al. 2019; Thomson and Beauchaine 2019). These frameworks are further supported by robust evidence with adolescents and adults, which has consistently found that higher baseline HRV relates to greater affective flexibility and abilities to modulate emotional expression (Demaree et al. 2004), as well as more efficient ER strategies (Fabes and Eisenberg 1997).

If low baseline HRV reflects vulnerability for poor emotion regulatory capacity, it may be presumed that children with lower baseline HRV have more difficulty achieving regulatory milestones. Achieving these milestones is critical as children enter formal schooling and are expected to regulate their emotions and behaviors in the classroom. School entry also represents an increased demand on children's social development with peers, which presents ER challenges unique from those in relationships with adults. For example, peers are more likely to assert their own needs and be less tolerant of violations of fairness than adults are (Engelmann and Tomasello 2019). Children must learn to regulate their emotions (e.g., frustration) to succeed in cooperative play and friendships (Blair et al. 2015). This skill begins to emerge in the preschool years and is cultivated by adult supervision and guidance, although this guidance begins to decline in elementary school when teachers are tasked with academic goals, and adult‐child ratios increase, granting children more social autonomy requiring more independent socioemotional skills (see Bierman 2011).

Consistent with this notion, some research has found positive associations between baseline HRV and social behaviors that succeed in ER in preschoolers, such as children's positive responses to play situations (e.g., positive affect; Calkins 1997). Similarly, low resting or baseline HRV has predicted increases in internalizing behaviors in children aged 3–6 years, as well as externalizing behaviors in elementary schoolers, both of which can have negative implications for peer relationships (Xu et al. 2014; Zhou and Buss 2021). Accordingly, low baseline HRV has been conceptualized as a socioemotional risk factor, whereas high baseline HRV has been viewed as universally beneficial. However, other work has failed to find links between baseline HRV and symptoms of emotion dysregulation in preschoolers and kindergarteners (Calkins et al. 2007; Fortunato et al. 2013; Hastings et al. 2008). Furthermore, some studies have reported inverse findings, where higher baseline HRV is associated with greater risk for dysregulated behavior (Hastings et al. 2000; Miller et al. 2017; Zahn‐Waxler et al. 1995). Taken altogether, there is reason to consider that baseline HRV may convey different psychological implications in studies with adults relative to those with children.

It may be possible that ongoing developmental processes interfere with the ability to detect trait‐level stability, explaining discrepant findings in young samples. Both ER and baseline HRV are developing throughout early and middle childhood. Studies of HRV in childhood (around ages 4–7) typically observe year‐over‐year increases in mean HRV levels (Dollar et al. 2020; Harteveld et al. 2021), suggesting that HRV may simply lack the stability to reflect an invariable trait at this age. A longitudinal assessment of children from kindergarten through second grade reported moderate rank‐order stability (*r*s = 0.47–0.59; Gatzke‐Kopp and Ram 2018). Although this degree of stability does not rule out the possibility that baseline HRV reflects meaningful individual differences in emotion regulatory capacity, it could also be possible that the unstable portion of HRV that is still developing over this time period has the greatest impact on ER outcomes. Research has not thoroughly examined factors that influence change in HRV over time, but exogenous factors likely play a key role. For instance, parental socialization of ER could help shape children's engagement of parasympathetic regulation over time (Zhang et al. 2020), just as coercive parental practices could shape dysregulated reactivity patterns (see Beauchaine and Gatzke‐Kopp 2012). Attending to the role of experiential context is likely significant in understanding how baseline HRV relates to young children's emerging ER outcomes.

### 1.2 Baseline HRV and Children's Sensitivity to Context

Reports of higher baseline HRV correlating with worse ER outcomes are fundamentally inconsistent with the ER framework extensively invoked in the literature. Such findings, however, would be consistent with the developmental sensitivity to context framework (Ellis et al. 2011). Developmental sensitivity models postulate that individuals differ in the extent to which they are affected by environmental influences (e.g., how sensitive they are to factors in the environment that shape development, such as parenting). Physiological markers that are thought to denote such sensitivity may, therefore, have conflicting patterns of correlation with developmental outcomes. For instance, children who are more sensitive to environmental factors will benefit greatly when raised in loving and enriched environments, but will suffer more consequences when raised in adverse conditions (Ellis et al. 2011). Although early models of environmental sensitivity (ES) have focused primarily on endocrine markers of stress reactivity as indices of sensitivity (i.e., cortisol), HRV may function in this capacity by facilitating greater engagement with and processing of environmental context (Thayer et al. 2009).

Differentiating these two frameworks, which we will hereon refer to as ER and ES, can be challenging. For instance, in positive environmental contexts, both models would predict better ER outcomes among children with higher baseline HRV (e.g., better abilities to regulate aggressive behavior among peers). However, in the context of negative or adverse environmental contexts, the ER framework would predict that higher baseline HRV would act as a buffer or protective factor, such that children with higher baseline HRV would have better regulatory competence than those with lower baseline HRV. In contrast, the ES framework would predict that children with higher baseline HRV would demonstrate a stronger correlation between environmental quality and regulatory competence because higher HRV reflects the extent to which the environment shapes development. In other words, children with higher baseline HRV would be expected to show *better* ER outcomes at the higher end of environmental quality and *worse* ER outcomes at the lower end of environmental quality. By comparison, children with lower baseline HRV would be expected to have comparable levels of ER outcomes across the range of environmental quality. Differentiating between these frameworks can be difficult, as there are conditions in which the same pattern of associations is predicted, despite each framework positing a different mechanistic pathway. For instance, in the context of a generally positive and emotionally supportive environment, both models would predict better socioemotional outcomes among children with higher HRV. The ER model would argue that higher HRV reflects an innate trait that is associated with individual differences in ER capacity, whereas the ES model would argue that higher HRV reflects a better ability to extract the positive guidance and emotional support, resulting in the development of better ER skills. Only in the context of the full range of environmental quality is the distinction between these frameworks evident. In particular, the ER framework argues that the innate regulatory capacity reflected by higher HRV would serve to buffer or protect children from worse socioemotional outcomes, whereas the ES framework would argue that children with higher HRV are more sensitive to the adversity and will, therefore, fare worst.

It is possible that both models offer valid insight into the role of HRV in supporting socioemotional development. Higher HRV may initially reflect an increased sensitivity to environmental influences, which serves to shape HRV into an indicator of ER capacity. A variety of studies have examined whether environmental context, typically family adversity, moderates associations between baseline HRV and children's psychopathology. Consistent with the ES framework, Skibo et al. (2020) found that, relative to those with lower HRV at rest, 18‐month‐olds with higher resting HRV had poorer regulation of anger at age 3.5 in the context of maternal insensitivity and better regulation in the context of maternal sensitivity. The ES framework was also partially supported by Eisenberg et al. (2012), who found that 54‐month‐olds with high baseline HRV had the lowest levels of aggression in high‐quality environments (i.e., families with higher income, education, and marital harmony) compared to those with lower baseline HRV, although no differences were found in lower‐quality environments. In contrast, several studies have reported findings more consistent with the ER framework, although the samples were often older. El‐Sheikh et al. (2001) found that children aged 8–12 (*M* = 9.90) with high baseline HRV had fewer concurrent externalizing and internalizing problems in the context of marital conflict relative to children with lower baseline HRV, suggesting that higher HRV served as a protective factor. Similar results have been found longitudinally, where higher baseline HRV at age 5 buffered the effect of marital hostility on children's behavioral outcomes measured 3 years later (Katz and Gottman 1995). Although there has been very limited longitudinal research on this topic, the existing evidence suggests that support for HRV as a marker of ER outcomes is not consistent until late childhood/early adolescence, whereas studies of younger children (infancy through age 6) are more likely to report no observed associations between baseline HRV and emotion regulatory outcomes, or to report adverse associations with higher baseline HRV. Therefore, it is possible that early to middle childhood represents a critical developmental phase wherein the psychological significance of HRV is shifting. The present study examines this possibility by examining children longitudinally across the first 3 years of elementary school (kindergarten through second grade) to determine whether there is a developmental shift in how baseline HRV and environmental quality relate to children's socioemotional functioning. Because school represents a critical social context for children's emerging ER and social skills, we focus on this environment.

### 1.3 School as a Developmental Context

Much of the research examining the impact of children's environment has focused on the home context, specifically parental influences. Consequently, relatively less research has attended to how the school context interacts with children's physiological functioning. School entry marks a major developmental transition distinct from precursory experiences that have shaped children's physiological functioning to date. Most children are also navigating new peer relationships upon kindergarten entry, which requires the ability to regulate emotions. Difficulties establishing appropriate social connections are associated with the development of psychopathology (Trentacosta and Shaw 2009). This is likely to reflect both the role that emotion dysregulation plays in disrupting social interactions and predisposing individuals to psychopathology, as well as the transactional risks associated with peer rejection. This suggests that successful peer relationships are an important indicator of children's socioemotional development.

Educational research indicates that teachers have an important role in shaping children's social development. Particularly for younger children, teachers are often perceived as social referents who enforce social climates and norms in the classroom, which can influence peer dynamics (see Farmer et al. 2011). Children may also take cues from teachers that influence their opinions of peers. For example, a child who receives high levels of teacher support may be rated as more liked by peers (Hughes et al. 2001). For children who enter school less socially prepared (e.g., more emotionally volatile) and at risk of peer problems like exclusion, teacher–child closeness (i.e., openness, affection, and warmth) may attenuate disruptive behavior problems and promote prosociality (Ladd and Burgess 2001; McCormick et al. 2014). Teacher–child closeness appears more reliably explained by teacher–specific factors (e.g., emotional status) than child‐specific factors (e.g., aggression), suggesting that closeness represents a socioemotional context independent of child effects (Choi and Dobbs‐Oates 2016). This contrasts with teacher–child conflict (i.e., anger and resistance between a teacher and child), which is commonly attributed to factors like child aggression and represents a component of the teacher–child relationship distinct from closeness (Choi and Dobbs‐Oates 2016; Pianta et al. 1995).

Teacher–child closeness may interact with children's baseline HRV to predict social outcomes. Applying the ER framework, low baseline HRV would be expected to denote vulnerability to peer problems, which would be exceptionally exacerbated in the context of low teacher–child closeness, whereas higher baseline HRV would be expected to serve a protective function in the context of low closeness. Thus, children with low baseline HRV would be in greatest need of teacher support. Conversely, the ES framework would postulate that children with higher baseline HRV are especially sensitive to environmental influences and thus would fare best in the context of high closeness but worst in the context of low closeness. Of the scant literature in the school context, some research has substantiated the ES framework with non‐physiological indicators of susceptibility. For example, children high in behavioral reactivity have displayed the best social skills and classroom adjustment in the context of high teacher–child closeness compared to children lower in behavioral reactivity (Baker 2006; Silver et al. 2005). According to the ES framework, children with higher baseline HRV would benefit most from teacher support since they are most receptive to aspects of the teacher–child relationship.

### 1.4 Current Study

**FIGURE 1:**
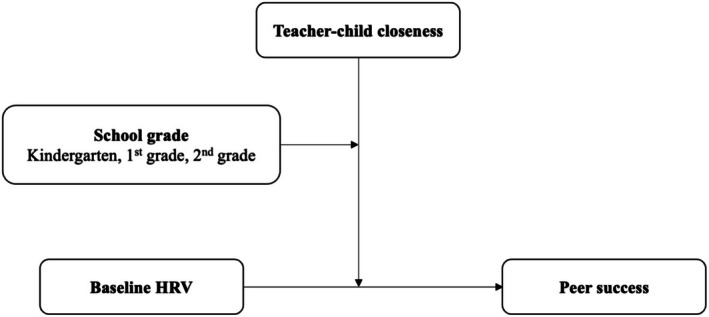
Conceptual model of the three‐way interaction among baseline HRV, teacher–child closeness, and school grade predicting peer success. Models were fit using FIML estimation. In kindergarten, baseline HRV and teacher–child closeness were assessed in the fall, and peer success was assessed in the spring. All data for first and second grade were collected in the spring.

This study examined the association between baseline HRV and teacher–child closeness in predicting children's social success to evaluate whether the pattern of association was more consistent with a model of baseline HRV as an index of ER or ES. We additionally employed three‐way interaction model (moderated moderation) to determine whether the pattern of association changed across the first 3 years of elementary school (see Figure 1 for a conceptual diagram). Given that associations between baseline HRV and socioemotional outcomes are less commonly reported in younger children (e.g., Eisenberg et al. 2012; Skibo et al. 2020), we hypothesized that earlier data would be consistent with the ES model, wherein the effect of teacher–child closeness on peer success would be strongest among children higher in baseline HRV (i.e., these children would show the best and worst peer outcomes as a function of teacher–child closeness). Additionally, given the influences of teacher relationships on children's social development become less prominent with age (Bierman 2011), children may be particularly susceptible to these relationships earlier in school. We therefore examined whether this association weakened over time, such that peer success was predicted by a main effect of higher baseline HRV, consistent with the ER framework and more commonly observed in older children (e.g., El‐Sheikh et al. 2001; Katz and Gottman 1995).

## 2 Method

### 2.1 Participants

Data were collected from children across 10 elementary schools in a lower socioeconomic status, urban school district in central Pennsylvania. This district served a community with higher rates of property and violent crime compared to the state overall (2 and 4.5 times as high, respectively). Most households were headed by a single woman (61%) and most caregivers had a high school education or less (79%). The majority of children in the school district qualified for free or reduced‐price lunches (86%) and a minority were considered proficient in math and reading skills (< 25%). The original study tested a multicomponent school‐based intervention for high‐risk children with behavior problems (Gatzke‐Kopp et al. 2012). Children were recruited based on a 10‐item screener adapted from the revised Teacher Observation of Child Adaptation (TOCA); (Werthamer‐Larsson et al. 1991), which teachers completed to assess levels of aggressive and oppositional‐defiant behaviors along a 6‐point scale for all children in their classrooms (*n* = 1192). Items included “This child yells at others,” and “This child breaks rules.” Higher scores indicated greater externalizing problems. Children with externalizing scores in the upper 25% of each classroom (*n* = 207) were recruited and randomly assigned to the control (*n* = 107) or intervention (*n* = 100) groups. An additional 132 children whose scores were in the bottom 25% of each classroom were recruited as a comparison group. Within‐classroom recruitment was done to ensure that teacher and classroom variables were not confounded with child group status. The full sample consisted of 339 children (*M*_age_ = 66.32 months, SD_age_ = 4.13 months, 64.3% male) with diverse racial‐ethnic backgrounds (70% Black, 20% Hispanic, 9% Caucasian, < 1% Asian). Across the full sample, TOCA scores were rated low‐moderate, although children were rated across the full range (*M* = 23.35, SD = 12.80, range = 10.00–60.00).

As described below, analyses incorporated data collected from (a) teacher self‐report, (b) classroom peer nominations, and (c) child psychophysiological assessments. Because data could be missing in one category and not another (e.g., teacher refusal to participate in the study), analyses used full information maximum likelihood (FIML) estimation to retain as much data as possible. All 339 children had at least one source of data in kindergarten. Between kindergarten and first grade, 38 children were lost from the study. These children did not differ from the 301 children who remained in the study regarding their symptom screening score at study entry (*t*[46.85] = 0.46, *p* = 0.65). An additional 28 children were lost between first and second grade. These children did significantly differ from the 273 children who remained in the study, such that children who were lost had higher behavior problems at study entry (*t*[31.05] = 2.39, *p* = 0.02). This pattern of attrition is consistent with the tendency to lose higher‐risk participants over the course of a longitudinal study. However, the full range of screener scores was still represented within and across school years. For breakdowns of the TOCA and demographics for children with any data within and across years, refer to Table 1.

**TABLE 1:** Child demographics and behavioral screener ratings.

	Kindergarten	First grade	Second grade	All grades
*N*	339	301	273	1017
Age				
*M* (SD)	5.63 (0.35)	5.64 (0.36)	5.64 (0.35)	5.63 (0.35)
Range	5.04–6.99	5.04–6.99	5.04–6.99	5.04–6.99
% Male	64.00	66.00	66.00	64.00
% Black	70.00	72.00	73.00	70.00
TOCA				
*M* (SD)	23.35 (12.80)	23.23 (12.82)	22.59 (12.45)	23.35 (12.79)
Range	10.00–60.00	10.00–60.00	10.00–60.00	10.00–60.00

*Note:* Age represents children's age in years in the fall of kindergarten. The minority of children who were “not Black” were categorized as either Caucasian, Hispanic, or Asian. Higher TOCA scores indicated more aggressive and oppositional behavior.

Abbreviation: TOCA, Teacher Observation of Child Adaptation (Werthamer‐Larsson et al. 1991).

All children received a universal socioemotional learning curriculum from the beginning of kindergarten to the end of first grade as a standard district curriculum (PATHs; Kusché and Greenberg 1994). Children in the intervention group underwent an additional “friendship group” component beginning in the spring of kindergarten. These groups occurred weekly through the first half of first grade and focused on self‐regulation skills and positive peer relationships (Bierman et al. 1996). However, no evidence of intervention effects has been found with regard to peer success or other behavioral outcomes, and thus the full sample is examined developmentally without consideration of group status.

### 2.2 Procedures

Protocols were approved by The Pennsylvania State University Institutional Review Board. Caregivers provided written consent at enrollment, and children provided verbal assent at the time of assessment. Two cohorts (2008 and 2009) of children were screened and recruited between October and December of their kindergarten year and followed annually through second grade. Health‐related exclusion criteria (e.g., cardiovascular and respiratory conditions) were not screened in the original study. Children underwent psychophysiological assessments during school hours. In kindergarten, these assessments took place about 4 months into the school year. In first and second grade, assessments took place in the second half of the school year. Families were made aware of children's scheduled dates. Assessments were completed by research assistants in a recreational vehicle on the school premises. The vehicle was decorated with a child‐friendly outer‐space theme. Children were escorted from their classrooms to the vehicle and, after providing assent, had seven electrodes placed on their chests to capture electrocardiogram (ECG) and impedance cardiography. Two additional electrodes for the purpose of assessing electrodermal activity and a 32‐channel electroencephalogram cap were placed on children, but those data are not examined here. Following equipment setup, children underwent a baseline assessment. Children watched a moving starfield and were told to imagine they were traveling through space to a distant planet where they would play a game and watch videos. Only the baseline data are examined here.

Teachers completed surveys on children's socioemotional, behavioral, and academic adjustment. Kindergarten teachers completed reports in January, and first and second grade teachers completed reports between March and April. Teachers were compensated with a $15 gift card upon survey completion. Peer data were collected in the spring of each school year. All children were asked to participate in the sociometric ratings unless their caregivers returned a signed refusal form.

### 2.3 Measures

#### 2.3.1 Baseline HRV

Children's baseline HRV was measured prior to any tasks during a 2‐min clip of a moving starfield. This type of neutral stimulus (vanilla baseline) is considered more developmentally appropriate than fixation baseline measures because young children often have difficulty remaining still and undistracted for several minutes. Having children watch a neutral film clip is thought to be beneficial in promoting attention and calmness, and has been done in previous studies with children of this age (e.g., Dollar et al. 2020). ECG data were collected using disposable, pre‐gelled cardiac electrodes placed on the distal right collarbone, and lower left and right ribs. An additional four electrodes were used to collect impedance cardiography. Mindware utilizes the raw impedance signal (Z0) to extract an estimate of respiratory frequency (see Ernst et al. 1999). Cardiac data were collected continuously at 500 Hz via the Biolab 2.4 acquisition system (Mindware, Westerville, OH). Trained research assistants reviewed the ECG data to identify and correct erroneous or missing beats due to movement artifact. HRV was calculated as the power in the respiratory frequency band 0.12–1.04 Hz in 30 s epochs (4 epochs for the 2‐min baseline task) via fast‐Fourier transform spectral analysis of the interbeat interval (IBI) time series obtained from the ECG. We used a wide bandpass (0.12–1.04 Hz) based on recommendations for filtering the IBI series per Allen et al. (2007) and Berntson et al. (1997) to accommodate the age range across the longitudinal assessments. If there were excessive artifacts in the signal such that more than two consecutive beats could not be visually identified, or the respiratory frequency during any epoch fell outside this range (< 1% of epochs), HRV was not scored for that epoch. Baseline values comprised the average of all available epochs. Descriptive analyses reported in Table 2 show that average baseline HRV increased slightly each year (*M*_K_ = 7.28, *M*_1_ = 7.41, *M*_2_ = 7.50). Baseline HRV was negatively correlated with age across years, reflecting an atypical age association (*r* = −0.10, *p* = 0.007; Dollar et al. 2020). This could be explained by some children having started kindergarten closer to 7 years old because they were less emotionally ready (age range = 5.04–6.99 years), and thus potentially lower in ER. Baseline HRV was moderately stable across years (*r*s = 0.47–0.59, *p*s < 0.001) and did not correlate with peer success or teacher–child closeness.

**TABLE 2:** Descriptives and correlations.

Variable	1	2	3	4	5	6	7	8	9	10	11	12	13	14	15	16	17	18	19	20	21	22	23	24
1. Gender	(1)																							
2. Race	−0.13*	(1)																						
3. Age	−0.01**	−0.05	(1)																					
4. Grade	−0.02	0.04	0.01	(1)																				
5. B respiration rate	−0.10*	0.03	0.05	0.01	(1)																			
6. B respiration rate (KF)	−0.09	0.02	0.09	—	—	(1)																		
7. B respiration rate (1S)	−0.06	0.09	0.13	—	—	0.23**	(1)																	
8. B respiration rate (2S)	−0.10	0.02	−0.07	—	—	0.08	0.29**	(1)																
9. TC conflict	−0.16**	0.13**	−0.03	0.04	0.09*	—	—	—	(1)															
10. TC conflict (KF)	−0.16**	0.14*	0.00	—	—	0.24**	0.03	0.05	—	(1)														
11. TC conflict (1S)	−0.18**	0.13*	0.04	—	—	0.26**	0.01	−0.05	—	0.62**	(1)													
12. TC conflict (2S)	−0.21**	0.15*	−0.05	—	—	0.16*	0.01	0.01	—	0.58**	0.65**	(1)												
13. TC closeness	0.21**	−0.09*	0.09*	−0.03	−0.06	—	—	—	−0.48**	—	—	—	(1)											
14. TC closeness (KF)	0.23**	−0.13*	0.17**	—	—	−0.18**	0.01	0.00	—	−0.47**	−0.29**	−0.34**	—	(1)										
15. TC closeness (1S)	0.24**	−0.13*	0.08	—	—	−0.09	0.07	−0.11	—	−0.32**	−0.46**	−0.32**	—	0.42**	(1)									
16. TC closeness (2S)	0.22**	−0.05	0.00	—	—	−0.12***	−0.03	−0.07	—	−0.28**	−0.30**	−0.56**	—	0.19**	0.34**	(1)								
17. Peer success	0.12**	−0.05	0.14**	−0.11**	−0.04	—	—	—	−0.47**	—	—	—	0.34**	—	—	—	(1)							
18. Peer success (KS)	0.07**	−0.08	0.14**	—	—	−0.08	−0.05	0.00	—	−0.43**	−0.39**	−0.37**	—	0.33**	0.24**	0.15**	—	(1)						
19. Peer success (1S)	0.12**	−0.02	0.10	—	—	−0.16*	−0.01	0.06	—	−0.48**	−0.49**	−0.38**	—	0.36**	0.35**	0.20**	—	0.47**	(1)					
20. Peer success (2S)	0.20**	0.00	0.13*	—	—	−0.11	0.02	0.00	—	−0.42**	−0.38**	−0.43**	—	0.30**	0.29**	0.32**	—	0.39**	0.52**	(1)				
21. B HRV	0.03	0.14**	−0.10**	0.01	−0.16**	—	—	—	−0.01	—	—	—	0.03	—	—	—	0.02	—	—	—	(1)			
22. B HRV (KF)	0.03	0.08	−0.12*	—	—	−0.22**	−0.10	−0.01	—	−0.03	0.01	−0.01	—	−0.02**	−0.01	0.05**	—	−0.08	0.11	0.10	—	(1)		
23. B HRV (1S)	0.06	0.18**	−0.10	—	—	−0.14*	−0.17**	0.01	—	−0.02	0.06	0.00	—	0.05*	0.00	0.02**	—	−0.06	0.05	0.08	—	0.59**	(1)	
24. B HRV (2S)	0.04	0.09	−0.13*	—	—	−0.08	0.09	−0.14*	—	−0.01	−0.12	−0.07	—	−0.03**	0.06	0.11**	—	0.02	0.09	0.04	—	0.47**	0.50**	(1)
*N*	339	339	339	681	681	286	262	241	681	301	271	239	681	301	271	239	681	336	270	243	681	286	262	241
*M*	0.36	0.70	5.63	0.91	21.12	21.78	20.97	20.63	2.27	2.26	2.24.	2.40	4.07	4.07	4.07	4.02**	0.17	0.19	0.16	0.11	7.40	7.28	7.41**	7.50.
SD	0.48	0.46	0.35.	0.81	3.52	3.32	3.83	3.66	1.19	1.15	1.18.	1.29	0.76	0.69	0.76	0.85**	0.37	0.34	0.38	0.39	1.30	1.29	1.31	1.27.
Range	0/1	0/1	5.04–6.99	0–2	10.13–35.01	14.72–34.58	10.13–40.86.	12.40–35.01.	1.00–5.00	1.00–4.88	1.00–5.00.	1.00–5.00.	1.12–5.00	1.88–5.00.	1.12–5.00.	1.50–5.00.	−0.94 to 1.00.	−0.71 to 1.00	−0.94 to 1.00.	−0.89 to 1.00.	2.49–10.36.	2.49–10.36.	3.63–10.27.	4.06–10.26.

*Note:* Gender was coded as 0 = male, 1 = female. Race was coded as 0 = not Black, 1 = Black. Age represents children's age in years in the fall of kindergarten. Indented variables represent grade‐specific measures, whereas non‐indented variables represent values across school years.

Abbreviations: 1S, first grade spring; 2S, second grade spring; B, baseline; KF, kindergarten fall; KS, kindergarten spring, TC, teacher–child.

* *p* < 0.05.

** *p* < 0.01.

*** *p* < 0.10.

#### 2.3.2 Teacher–Child Closeness

Teachers reported on closeness with their students using the “closeness” subscale of the Student‐Teacher Relationship Scale (STRS; Pianta 2001). The scale consisted of 8 items rated along a 5‐point Likert scale from 1 (*Definitely does not apply*) to 5 (*Definitely applies*). One item reads, “I share an affectionate, warm relationship with this child.” Items were averaged to create a summary score, with higher scores indicating closer teacher–child relationships. The subscale demonstrated acceptable reliability each year (*α*s = 0.88–0.92).

Children were generally rated as having moderate‐high teacher–child closeness each year, although closeness was also widely distributed (*M* = 4.02–4.07, SD = 0.69–0.85, range = 1.12–5.00), as shown in Table 2. Teacher–child closeness was moderately stable from kindergarten to first grade (*r* = 0.42, *p* < 0.001) and from first grade to second grade (*r* = 0.34, *p* < 0.001), and lower but still significantly correlated from kindergarten to second grade (*r* = 0.19, *p* = 0.01). Note that these associations reflect closeness as a child‐level trait, given that teachers differed across grades. Lastly, children with higher teacher–child closeness had higher peer success on average each year (*r*s = 0.32–0.35, *p*s < 0.001).

#### 2.3.3 Peer Success

Children were presented with a classroom roster with classmate photos and three cartoon faces to indicate how children felt about each classmate: a smiling face to indicate they “liked to play a lot” with a peer, a neutral face to indicate they “liked to play sometimes” with that peer, and a sad face to indicate they “did not like to play” with that peer. Children were first asked whether they knew each classmate, and if they did not know a classmate, that classmate was skipped. Percentages representing proportions of children who reported liking to play with and disliking to play with each child were calculated (i.e., the number of students who said they liked to play with each child divided by the total number of students in the class who provided ratings). For current analyses, a continuous social preference score was created by subtracting the proportion of “does not like to play” from “like to play” scores (i.e., social preference = like proportion − dislike proportion). Positive social preference scores indicated that more peers liked playing with the child than disliked doing so, whereas negative scores indicated that more peers disliked playing with the child. Scores could fall between −1.00 (100% dislike) and 1.00 (100% like). This calculation is based on previous work examining peer ratings and sociometric status (Coie et al. 1982). Similar measures have achieved satisfactory test–retest reliability (Asher et al. 1979) and have been shown to predict behavioral outcomes like aggression several years after kindergarten (Dodge et al. 2003). In our own data, sociometric ratings in kindergarten correlated with externalizing symptoms at the end of first and second grade, such that children with lower social preference scores had more behavior problems (*r*s = −0.33 and −0.40, respectively, *p*s < 0.001).

On average, children demonstrated moderate peer success such that they netted more positive nominations than negative ones, although levels decreased each year (*M*_K_ = 0.19, *M*_1_ = 0.16, *M*_2_ = 0.11), which was reflected in the negative correlation between grade and overall peer success (*r* = −0.11, *p* = 0.006). Within each year, the sample scores for peer success were observed across nearly the full continuum (range = −0.94 to 1.00). Peer success demonstrated moderate rank order stability across years, indicating that children rated higher in peer success tended to be rated similarly across years (*r*s = 0.39–0.52, *p*s < 0.001). Refer to Table 2 for more descriptives.

#### 2.3.4 Covariates

Given this sample was at risk of behavior problems, we controlled for teachers' perceptions of children's externalizing and emotional tendencies each year using the 8‐item “conflict” subscale of the STRS. For a full list of these items, see Table 3. Like closeness items, conflict items were measured along a 5‐point Likert scale from 1 (*Definitely does not apply*) to 5 (*Definitely applies*), and a composite score was created by averaging the eight items. Items were reverse coded such that higher scores indicated higher conflict. The subscale demonstrated high reliability each year (*α*s = 0.94–0.95). Controlling for teacher–child conflict was justified given its correlation with other subscales assessing aggression, oppositionality, and emotion dysregulation in this sample (e.g., *r*s = 0.87, 0.71, and 0.48 in kindergarten, respectively), as well as evidence that this subscale assesses negative aspects of the teacher–child relationship specifically evoked by the child (Choi and Dobbs‐Oates 2016). In the current sample, teachers rated children as having moderate teacher–child conflict on average each year, although conflict was widely distributed (*M* = 2.24–2.40, SD = 1.15–1.29, range = 1.00–5.00). Teacher–child closeness and conflict were negatively associated each year (*r*s = −0.46 to 0.56, *p*s < 0.001), which was expected given they were within‐teacher measures. These associations were moderate in strength, supporting that the conflict and closeness subscales were separate dimensions rather than opposite ends of the same spectrum.

**TABLE 3:** Teacher–child conflict items from the student‐teacher relationship scale (Pianta 2001).

Item	Content	Definitely does not apply	Does not really apply	Neutral or not sure	Applies somewhat	Definitely applies
2.	This child and I always seem to be struggling with each other	1	2	3	4	5
7.	This child easily becomes angry with me	1	2	3	4	5
8.	This child feels that I treat him/her unfairly	1	2	3	4	5
10.	This child remains angry or is resistant after being disciplined	1	2	3	4	5
11.	Dealing with this child drains my energy	1	2	3	4	5
12.	When this child is in a bad mood, I know we're in for a long and difficult day	1	2	3	4	5
13.	This child's feelings toward me can be unpredictable or can change suddenly	1	2	3	4	5
14.	This child is sneaky or manipulative with me	1	2	3	4	5

Children's age at the kindergarten screener (to control for differences in age at school entry), gender (0 = male, 1 = female), race (0 = not Black, 1 = Black), and baseline respiration rate were also included as covariates due to revelations of preliminary analyses (see Table 2). Given the small proportion of children not identified as Black, race was re‐coded as “Black” and “Other.” Overall, teachers rated higher levels of closeness and lower levels of conflict with girls relative to boys. There was also a small but significant tendency for teachers to rate higher levels of closeness and lower levels of conflict with non‐Black students as opposed to Black students. Black children had higher baseline HRV in first grade. Relative age within grade did not have an impact on teacher ratings of closeness beyond kindergarten, where older children tended to be rated higher in closeness, and had no association with conflict. Peers showed a slight tendency to rate girls and relatively older children more positively, although there was no effect of race on children's social success with peers.

Baseline respiration rate decreased each year (*M*_K_ = 21.78, *M*_1_ = 20.97, *M*_2_ = 20.63), paralleling developmental shifts in respiration (Shader et al. 2018), although it positively correlated with age in first grade (*r* = 0.13, *p* = 0.03). Children with higher baseline respiration rates tended to have lower baseline HRV, and greater teacher–child conflict, and lower teacher–child closeness in kindergarten. Lastly, respiratory rate was stable from kindergarten to first grade (*r* = 0.23, *p* = 0.0006) and first grade to second grade (*r* = 0.29, *p* < 0.0001), but not from kindergarten to second grade, demonstrating a weakening of this relation across more time. We did not control for other physical variables like height or weight.

### 2.4 Data Analytic Plan

Between‐teacher variances were low for kindergarten teacher–child closeness (16.38%), suggesting that students in different classrooms were rated similarly compared to students in the same classroom, and that a simple regression framework was justified over multilevel modeling. For this reason, we fit a linear regression model using the lavaan package in RStudio (Rosseel 2012; RStudio Team 2021). We appended data for kindergarten, first grade, and second grade to create a full dataset of *N* = 681. All continuous predictors were mean‐centered. Children's gender, race, age in kindergarten, teacher–child conflict, and baseline respiration rate were included as control variables in the model. To account for the nesting in the data, we used robust standard errors (McNeish et al. 2017). The following equation for modeling peer success is as follows:

$$\begin{array}{c} {\text{Peer Success}}_{i}=\beta_{0}+\beta_{1}\left(\text{gender}\right)+\beta_{2}\left(\text{race}\right)+\beta_{3}\left({\mathrm{age}}_{K}\right)+\beta_{4}\left(\text{conflict}\right)\\ \;+\beta_{5}\left(\text{baseline respiration}\right)+\beta_{6}\left(\text{baseline}\;\mathrm{HRV}\right)+\beta_{7}\left(\text{closeness}\right)\\ \;+\beta_{8}\left(\text{grade}\right)+\beta_{9}\left(\text{baseline}\;\mathrm{HRV}\times \text{closeness}\right)\\ \;+\beta_{10}\left(\text{baseline}\;\mathrm{HRV}\times \text{grade}\right)+\beta_{11}\left(\text{closeness}\times \text{grade}\right)\\ \;+\beta_{12}\left(\text{baseline}\;\mathrm{HRV}\times \text{closeness}\times \text{grade}\right)+\varepsilon_{i} \end{array}$$

$\beta_{0}$ represents the predicted value of peer success when all independent variables are zero (i.e., among male, non‐Black kindergarten students of average physiological and teacher–child relationship values) and $\varepsilon_{i}$ represents the error term for individual *i*. In all subsequent analyses, we used FIML estimation to account for missing data.

## 3 Results

### 3.1 Three‐Way Interaction

Results for the three‐way interaction are presented in Table 4. The model fit was significant (*χ* ^2^[12] = 214.78, *p* < 0.001) and explained about 24% of the variance in peer success across school years (adjusted *R* ^2^ = 0.24, *p* < 0.001). The intercept was significant, suggesting that when all other parameters were zero, predicted peer success was above average (*b =* 0.42, *p* = 0.000). Children with greater teacher–child conflict had lower peer success (*b* = −0.39, *p* = 0.000). Although children's age at the kindergarten screener predicted greater peer success (*b* = 0.12, *p* = 0.001), peer success tended to decrease across school years, which reflected the year‐to‐year mean decreases observed in peer success (*b* = −0.09, *p* = 0.004). There were no main effects of baseline HRV on peer success across school years, in contradiction to the ER framework. However, there was a main effect of teacher–child closeness across years such that children closer with their teachers tended to be more socially successful (*b* = 0.13, *p* = 0.02). There was a significant interaction between baseline HRV and teacher–child closeness (*b* = 0.10, *p* = 0.04). This was qualified by the three‐way interaction between baseline HRV, teacher–child closeness, and grade, which was borderline significant (*b* = −0.09, *p* = 0.06). Because the interaction with grade was a primary research objective, the interaction was probed further.

**TABLE 4:** Baseline HRV, teacher–child closeness, and school grade three‐way interaction predicting peer success.

Outcome	Predictor	*b*	*z*	*p*	95% CI (lower, upper)
Peer success	Intercept	0.42**	5.96	0.000	0.10, 0.21
	Gender	0.04	1.38	0.17	−0.01, 0.08
	Race	0.05	1.37	0.17	−0.02, 0.09
	Age	0.12**	3.39	0.001	0.05, 0.19
	TC conflict	−0.39**	−10.10	0.000	−0.14, −0.09
	B respiration rate	0.01	0.28	0.78	−0.01, 0.01
	B HRV	−0.04	−0.85	0.40	−0.04, 0.01
	TC closeness	0.13*	2.34	0.02	0.01, 0.11
	Grade	−0.09**	−2.86	0.004	−0.07, −0.01
	B HRV × TC closeness	0.10*	2.10	0.04	0.00, 0.07
	B HRV × Grade	0.06	1.24	0.22	−0.01, 0.03
	TC closeness × Grade	−0.01	−0.18	0.86	−0.04, 0.04
	B HRV × TC closeness × Grade	−0.09***	−1.86	0.06	−0.05, 0.00

*Note:* We used FIML estimation and robust standard errors; betas are standardized. Age represents children's age in years in the fall of kindergarten. Grade includes kindergarten, first grade, and second grade.

Abbreviations: B, baseline; TC, teacher–child.

* *p* < 0.05.

** *p* < 0.01.

*** *p* < 0.10.

### 3.2 Baseline HRV and Teacher–Child Closeness Interactions by Grade

To probe the three‐way interaction, we parsed associations by grade. Results are shown in Table 5. The overall model demonstrated good fit (*χ* ^2^[24] = 220.09, *p* < 0.001). The kindergarten model explained about 25% of the variance in kindergarten peer success (adjusted *R* ^2^ = 0.25, *p* < 0.001). Children rated their peers quite favorably on average (*b* = 0.57, *p* = 0.000). There were also main effects of covariates on peer success, such that older children and children with less teacher–child conflict had higher peer success (bs = 0.12 and −0.38, respectively, *p*s < 0.04). There was a borderline main effect of teacher closeness such that children who were closer with their teachers had greater peer success (*b* = 0.13, *p* = 0.05). There was no main effect of baseline HRV. The interaction between baseline HRV and teacher–child closeness was significant (*b* = 0.13, *p* = 0.04).

**TABLE 5:** Baseline HRV and teacher–child closeness 2‐way interactions predicting peer success by grade.

Outcome	Predictor	*b*	*z*	*p*	95% CI (lower, upper)
		Kindergarten			
Peer success	Intercept	0.57**	5.56	0.000	0.12, 0.26
	Gender	−0.00	−0.08	0.94	−0.07, 0.06
	Race	−0.00	−0.03	0.97	−0.07, 0.07
	Age	0.12*	2.13	0.03	0.01, 0.22
	TC conflict	−0.38**	−6.28	0.000	−0.14, −0.08
	B respiration rate	0.01	0.17	0.86	−0.01, 0.01
	B HRV	−0.08	−1.49	0.14	−0.05, 0.01
	TC closeness	0.13***	1.94	0.05	−0.00, 0.12
	B HRV × TC closeness	0.13*	2.06	0.04	0.00, 0.09
		First grade			
Peer success	Intercept	0.30**	2.77	0.006	0.03, 0.19
	Gender	0.02	0.39	0.70	−0.07, 0.10
	Race	0.06	1.08	0.28	−0.04, 14
	Age	0.12***	1.81	0.07	−0.01, 0.26
	TC conflict	−0.45**	−7.06	0.000	−0.18, −0.10
	B respiration rate	−0.02	−0.31	0.76	−0.01, 0.01
	B HRV	0.09***	1.69	0.09	−0.00, 0.06
	TC closeness	0.13*	2.07	0.04	0.00, 0.13
	B HRV × TC closeness	0.01	0.22	0.82	−0.04, 0.05
		Second grade			
Peer success	Intercept	0.06	0.42	0.68	−0.08, 0.12
	Gender	0.12*	2.01	0.04	0.03, 0.19
	Race	0.09	1.37	0.17	−0.03, 0.19
	Age	0.12*	2.12	0.03	0.01, 0.26
	TC conflict	−0.35**	−4.58	0.000	−0.15, −0.06
	B respiration rate	0.03	0.47	0.64	−0.01, 0.02
	B HRV	0.01	0.15	0.88	−0.03, 0.04
	TC closeness	0.10	1.32	0.19	−0.02, 0.11
	B HRV × TC closeness	−0.03	−0.67	0.50	−0.05, 0.02

*Note:* We used FIML estimation and robust standard errors; betas are standardized. Age represents children's age in years in the fall of kindergarten. Grade includes kindergarten, first grade, and second grade.

Abbreviations: B, baseline; TC, teacher–child.

* *p* < 0.05.

** *p* < 0.01.

*** *p* < 0.10.

The first grade model explained about 28% of the variance in first grade peer success (adjusted *R* ^2^ = 0.28, *p* < 0.001). Children continued to rate peers approvingly, although at a lower level than kindergarten (*b* = 0.30, *p* = 0.006), and peer success was lower among children with greater teacher conflict (*b* = −0.45, *p* = 0.000). Children with greater teacher–child closeness were more successful with peers in first grade (*b* = 0.13, *p* = 0.04), and older children were only marginally so (*b* = 0.12, *p* = 0.07). Contrary to the kindergarten model, the interaction between baseline HRV and teacher closeness was not significant. There was a positive association between higher baseline HRV and greater peer success consistent with the ER framework, but this did not reach significance (*b* = 0.09, *p* = 0.09).

Lastly, the second grade model explained about 22% of the variance in second grade peer success (adjusted *R* ^2^ = 0.22, *p* < 0.001), but like first grade, did not show a significant interaction between baseline HRV and teacher–child closeness. The marginal effect of baseline HRV was also lost, as was the main effect of teacher–child closeness. Only age and teacher–child conflict associated with peer success in directions reflected in previous models (bs = 0.12 and −0.35, respectively, *p*s < 0.03). Girls were more successful with peers in second grade (*b* = 0.12, *p* = 0.04).

### 3.3 Simple Slopes Analyses

**FIGURE 2:**
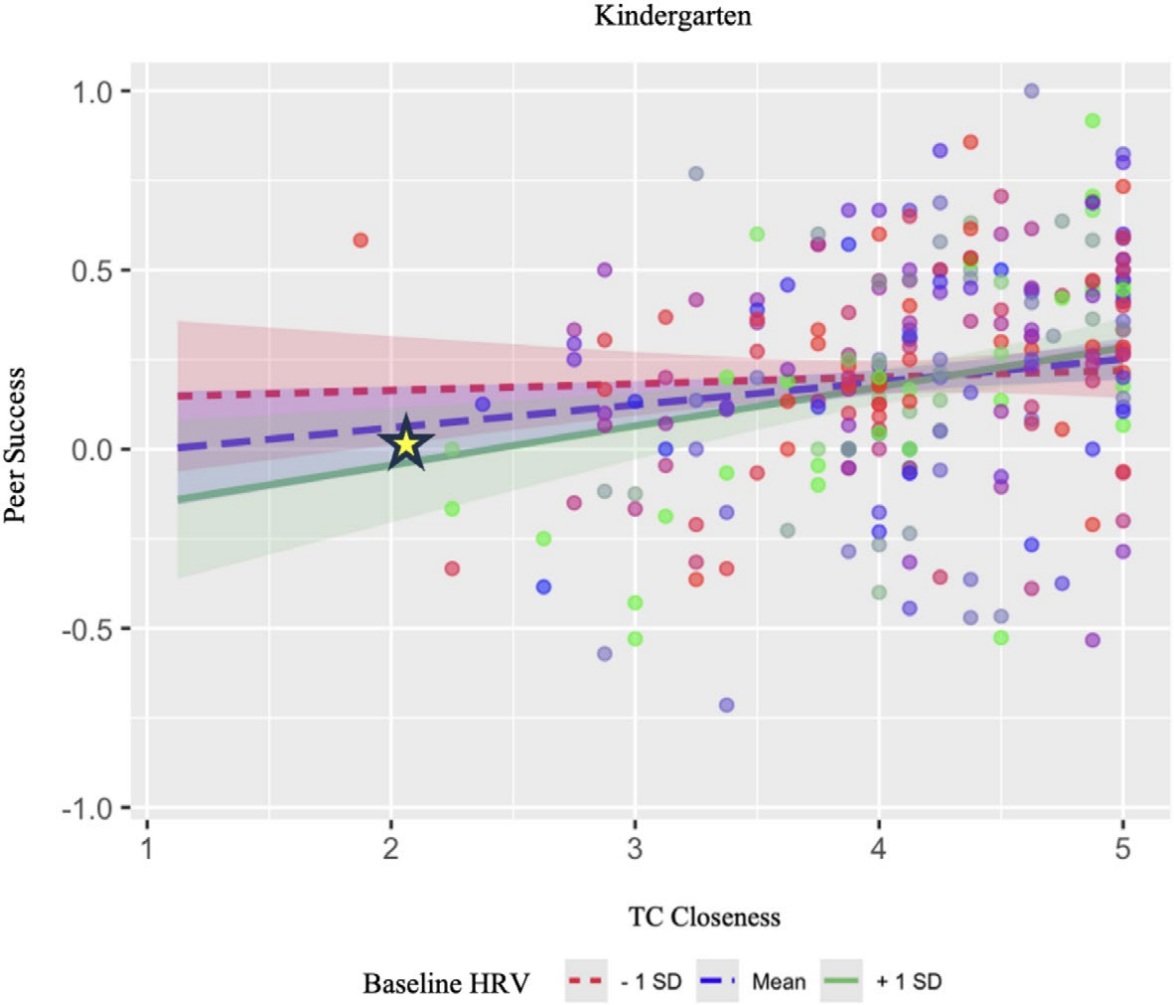
Simple slopes plot depicting the interaction between baseline HRV and teacher–child closeness predicting peer success in kindergarten. TC, teacher–child. Teacher–child closeness was rated along a 5‐point scale where higher scores indicated higher closeness. Peer success was assessed using social preferences scores that ranged from −1.00 (100% of peers disliked the child) to 1.00 (100% of peers liked the child). The yellow star next to the green and dotted blue lines (+ 1 SD and mean baseline HRV) represents the significant associations between high and average baseline HRV and low peer success in the context of lower kindergarten teacher–child closeness. Variables were not mean centered for this plot.

To explore the significant interaction between baseline HRV and teacher–child closeness in kindergarten, we probed teacher–child closeness slopes at ±1 SD of baseline HRV using the semTools package (Jorgensen et al. 2022). See Table 6 for kindergarten simple slopes output. We found the slope of teacher–child closeness predicting peer success was significant at high levels of baseline HRV (*b* = 0.10, *p* = 0.001) and average levels of baseline HRV (*b* = 0.06, *p* = 0.02) but not low levels. That is, children with high and average levels of baseline HRV had lower peer success in the context of low teacher–child closeness, which partially supported an ES framework. In other words, children with high and average baseline HRV were more sensitive to the effects of teacher–child closeness, faring worse when closeness was low. To visually analyze the simple slopes in kindergarten, we plotted the interaction using the jtools package (see Figure 2; Long 2022).

**TABLE 6:** Simple slopes of teacher–child closeness at ±1 standard deviation of baseline HRV in kindergarten.

B HRV levels	TC closeness simple slopes			
	*b*	*z*	*p*	95% CI (lower, upper)
−1 SD	0.02	0.65	0.52	−0.04, 0.09
Mean	0.06*	2.34	0.02	0.01, 0.11
+1 SD	0.10**	3.41	0.001	0.04, 0.15

*Note:* We used FIML estimation and robust standard errors; betas are standardized.

Abbreviations: B, baseline; TC, teacher–child.

* *p* < 0.05.

** *p* < 0.01.

## 4 Discussion

The current study tested a three‐way interaction among baseline HRV, teacher–child closeness, and grade to examine changes in the function of baseline HRV across school years. We were specifically interested in whether patterns emerged to support baseline HRV as an ES indicator, such that the implications of baseline HRV on peer success (an antecedent of psychopathology during this developmental period) would be dependent on teacher–child closeness, or as an ER indicator, such that baseline HRV would positively predict social success. The main finding was that associations between baseline HRV and peer success differed across early school years—specifically, support for the ES framework was found in kindergarten, whereas first grade findings trended toward supporting the ER framework, although results only approached conventional levels of significance. Neither framework was supported in second grade. Thus, within the same participants, different models of baseline HRV emerged across school years. These findings illustrate the importance of considering developmental processes in psychophysiological research with children.

The interaction between baseline HRV and teacher–child closeness in kindergarten was partially consistent with the ES framework. Children with high and average levels of baseline HRV appeared to be more sensitive to the influence of teacher closeness (or lack thereof) on their social success with peers, whereas children with low baseline HRV did not seem to be affected by teacher closeness. This finding supports literature suggesting higher resting or baseline HRV acts as a sensitivity factor in the context of adversity (Eisenberg et al. 2012; Skibo et al. 2020). However, while these results deviate from the ER framework in which higher baseline HRV is considered to be invariably beneficial, results did not indicate that kindergarteners higher in baseline HRV had significantly better social success in the context of greater teacher closeness, as would be expected in the full ES framework (Boyce and Ellis 2005).

Testing the ES framework can be extremely difficult, as it requires the assumption that the full range of “environmental quality” has been sampled, without theoretical clarification on what qualifies as “environment.” In the present study, we focused on teacher–child closeness as one aspect of the environment because of its proximal relation and theoretical role in guiding children's social success, and indeed our sample included the full range of variance on this measure. However, children are influenced by all aspects of their environment, including socioeconomic and neighborhood factors as well as parenting quality. Given that environments are rarely uniformly “good” or “bad,” but rather contain a mixture of risk and protective factors, it is not clear how best to conceptualize “environment.” The present sample was drawn from a demographically high‐adversity region, and as such, may not have reflected the full range of environmental quality on the enriched end. In other words, even among those with the highest levels of teacher closeness, exposure to other sources of adversity may have limited the extent to which the hypothesized advantages of higher baseline HRV predicted in the ES model could be observed. Our findings are at least somewhat consistent with a recent study that examined the association between baseline HRV and indices of prosocial behavior across three different samples of preschool‐aged children. Results across all three samples revealed a curvilinear effect, whereby moderate levels of HRV were associated with the highest levels of prosocial behavior, with lower levels of prosocial behavior observed at the highest and lowest levels of HRV (Miller et al. 2017). Although all three samples were drawn from upper‐middle‐class, low‐risk populations, it appears that children with the highest HRV may be more susceptible to the influence of poor relationships or other adverse circumstances, even in the context of generally low risk. However, this pattern may be specific to younger children, as the ES model was only supported in the current sample for children in kindergarten.

In the first‐grade model, results gravitated toward supporting the ER framework. Although we must interpret the borderline significant relation between baseline HRV and peer success in first grade with caution, this finding is most consistent with Polyvagal Theory and work arguing that low baseline HRV denotes risk of maladjustment, whereas higher HRV can serve as a protective factor (Porges 2007). Another way to conceptualize this finding is that higher baseline HRV trended toward acting as a buffer of the association between low teacher–child closeness and low peer success. The absence of the association between baseline HRV and peer success in kindergarten is consistent with other studies that have failed to detect associations between baseline HRV and behavioral indices of emotion regulatory capacity in younger children (e.g., Calkins et al. 2007; Fortunato et al. 2013; Hastings et al. 2008). This pattern of findings across the literature indicates that this association may emerge around age 7, suggesting that baseline HRV does not reliably function as an endophenotypic marker of vulnerability for psychopathological symptoms prior to this age, nor does it act as a strong buffering influence on associations with socioemotional problems. However, support for this hypothesis would be much stronger had the effect been fully significant in first grade and continued to manifest in second grade. The failure to replicate a sustained effect of baseline HRV in second grade, even if marginal, may be a function of the social and environmental measures of focus. We were particularly interested in peer success as a measure of children's ER skills because it directly measured a functional and meaningful developmental outcome (as opposed to an adult‐rated symptom checklist). However, this particular measure may not be as effective in this context. While children typically encounter new teachers each year, they are usually exposed to the same broad set of peers. Classroom compositions change from year to year, but with each year students are likely accumulating familiarity with their peers. As such, peer success in the first year may reflect children's ability to make new friends in a novel environment, but over time children may establish more stable friendships and it may be less sensitive as a measure of ER skills. This idea is supported by the descriptive data that rank‐order stability increased across years, but the net peer success scores decreased on average, suggesting that children were refining smaller but more stable social networks.

### 4.1 Strengths and Limitations

This study adds to a limited amount of psychophysiological research that examines children longitudinally. While many studies focus on questions of stability and reliability in physiological markers, this study further considers whether the psychological implications of physiological markers are stable. HRV was moderately stable across years (*r*s = 0.47–0.59), providing some support for HRV as a trait‐level index. However, the psychological correlates of HRV changed across years. These findings suggest that this childhood period may represent an important developmental stage, capturing critical transitions in the development of ER.

This study is among few studies that investigate interactions with baseline HRV in the school context rather than family or early childcare settings. We were also able to incorporate multiple informants in the school context, eliminating bias associated with similar reporters. Sociometric ratings provided valuable insight into children's peer success compared to teacher reports. Teacher and peer reports of similar constructs are not always highly correlated (e.g., Fry and Gatzke‐Kopp 2021), perhaps because they assess slightly different constructs. For example, teachers are more attuned to children's abilities to regulate during structured activities, whereas peers are more attuned to children's skills in social contexts not directly observed by teachers (e.g., recess). Thus, sociometric reports may not only provide greater perspective into peer success, but also increase the validity and accuracy of peer‐specific outcomes.

It is important to note that findings regarding “high” and “low” HRV are sample specific, and there are no normed thresholds for classifying HRV, making comparisons across studies more challenging. It has been argued that the same values of HRV could be considered “high” in one study and “low” in another depending on the sampling (Obradović 2012). To the extent that environmental exposures over the first 5 years of life impact children's HRV levels, children in the present sample may have a more truncated representation of the physiological range of HRV. In other words, we may have failed to detect an association between high or average HRV and better peer success if the children in the sample did not have “high” or “average” HRV relative to other typically developing children of this age. That said, sample means in the current study are comparable to those in other low‐risk samples (e.g., Miller et al. 2017).

We must note limitations. This sample was high‐risk, with families from lower socioeconomic status communities, higher rates of crime, and children largely of racial/ethnic minority status. Although focusing on high‐risk children is extremely crucial given their increased vulnerability to socioemotional challenges, future research should examine whether findings are generalizable to lower‐risk samples. Additionally, results are correlational and do not provide information on the temporal precedence of teacher–child closeness and baseline HRV (or peer success in first and second grade). We must also acknowledge selective attrition from first to second grade. Children who attritted between these years tended to score worse on the kindergarten screener assessing externalizing problems. It is unclear whether unique characteristics of children who were lost would impact the nature of the results in second grade.

Final considerations relate to our method. First, we did not control for physical variables in our analyses (e.g., children's height and weight). Although we were unable to collect these variables to keep our school procedures efficient and developmentally appropriate, such variables tend to be closely associated with age during this developmental period and may have correlated with cardiorespiratory parameters we assessed, introducing noise in our analyses. Second, our baseline assessment of HRV, which consisted of a 2‐min moving starfield video, was not a fixation baseline. Vanilla baselines are thought to be beneficial in maintaining the attention of younger participants who may have a difficult time sitting still and are frequently used in research with children (e.g., Calkins et al. 2007; Dollar et al. 2020; Hastings et al. 2008). However, attentional engagement may result in a reduction in HRV relative to a purely resting baseline (Jennings et al. 1992). Providing an attentional task (such as asking all participants to imagine traveling to space) may also constrain variance by having all participants engaged in a common activity, unlike fixation baselines, which may maximize individual differences by allowing participant minds to wander. It is possible that the limited support for the ER framework is a function of the possible constraints on between‐participant variance in HRV that may have been more evident in a purely resting baseline.

### 4.2 Future Directions

Future research may consider other developmental processes involved in children's peer outcomes and socioemotional adjustment more broadly. ER is rooted in a multitude of psychophysiological processes, and incorporating other factors (e.g., electrodermal activity or prefrontal control) may expand our mechanistic understanding of how physiology influences behavior, as well as further inform sensitive periods for affective regulation and environmental responsiveness. Additionally, given that both teacher–child closeness and baseline HRV are associated with peer outcomes, findings could inform early childhood preventions and interventions to reduce children's exposure and vulnerability to stressors, as well as promote resilience and wellbeing. For example, kindergarten teachers may consider how they assist children in forming relationships with new peers, especially children who appear well‐regulated. As children get older, it may become important to focus on children who appear more dysregulated, as they may be at the highest risk of peer problems. Implementing programs before the start of kindergarten may also be effective in reducing the amount of stress and dysfunction associated with the transition to kindergarten. Customizing approaches to specific children depending on their needs and environmental responses may be particularly beneficial, although not currently feasible in most educational settings. Future work could assess classroom climates more generally, as adjusting classrooms to address the broader needs of children may be more practical than instruction on a child‐by‐child basis (Roubinov et al. 2020). Future work may also consider assessing predictors of high‐quality teacher–child relationships, as this may inform how to best train teachers to promote close relationships with their students and, in turn, their students' healthy adjustment.

## 5 Conclusions

This study found unique models of baseline HRV and peer success across early school years. Average‐to‐high baseline HRV was negative for peer success in kindergarten in the context of low teacher–child closeness, partially supporting the ES framework. However, high baseline HRV was borderline significant in predicting peer success in first grade regardless of teacher–child closeness levels, cautiously supporting the ER framework. Results suggest that the effects of baseline HRV should be considered within a developmental context. This study expands prior research that has utilized mostly teacher reports of social functioning and contributes to literature on person‐environment interactions in the underrepresented school context. Findings have implications for future research related to HRV, social adjustment, and person‐environment interactions across development.

## Author Contributions

**Danielle R. Rice:** conceptualization, formal analysis, visualization, writing – original draft. **Lisa M. Gatzke‐Kopp:** conceptualization, investigation, resources, supervision, writing – review and editing. **Zachary Fisher:** formal analysis, methodology, supervision, writing – review and editing.

## Conflicts of Interest

The authors declare no conflicts of interest.

## Data Availability

Data and analytic code for this study are available upon request to the corresponding author, DRR.
